# Neural Processing of Emotional Prosody across the Adult Lifespan

**DOI:** 10.1155/2015/590216

**Published:** 2015-10-25

**Authors:** Liliana Ramona Demenescu, Yutaka Kato, Klaus Mathiak

**Affiliations:** ^1^Department of Psychiatry, Psychotherapy and Psychosomatics, University Hospital Aachen, RWTH Aachen, 52074 Aachen, Germany; ^2^Clinical Affective Neuroimaging Laboratory, 39120 Magdeburg, Germany; ^3^Department of Neurology, Otto von Guericke University of Magdeburg, 39120 Magdeburg, Germany; ^4^Leibniz Institute for Neurobiology, 39118 Magdeburg, Germany; ^5^Tsutsuji Mental Hospital, Tatebayashi, Gunma Prefecture, Japan; ^6^JARA-Translational Brain Medicine, 52074 Aachen, Germany

## Abstract

Emotion recognition deficits emerge with the increasing age, in particular, a decline in the identification of sadness. However, little is known about the age-related changes of emotion processing in sensory, affective, and executive brain areas. This functional magnetic resonance imaging (fMRI) study investigated neural correlates of auditory processing of prosody across adult lifespan. Unattended detection of emotional prosody changes was assessed in 21 young (age range: 18–35 years), 19 middle-aged (age range: 36–55 years), and 15 older (age range: 56–75 years) adults. Pseudowords uttered with neutral prosody were standards in an oddball paradigm with angry, sad, happy, and gender deviants (total 20% deviants). Changes in emotional prosody and voice gender elicited bilateral superior temporal gyri (STG) responses reflecting automatic encoding of prosody. At the right STG, responses to sad deviants decreased linearly with age, whereas happy events exhibited a nonlinear relationship. In contrast to behavioral data, no age by sex interaction emerged on the neural networks. The aging decline of emotion processing of prosodic cues emerges already at an early automatic stage of information processing at the level of the auditory cortex. However, top-down modulation may lead to an additional perceptional bias, for example, towards positive stimuli, and may depend on context factors such as the listener's sex.

## 1. Introduction

During adulthood, emotion recognition ability declines with advancing age. This process is independent of stimulus modality, that is, visual, auditory, and bodily expression modalities [1–5]. The decline is more pronounced for negative emotions, while the ability to discriminate positive emotions was preserved over age [6]. The neural correlates of this aging process and contributions from sensory processes are little known.

Only few studies examined age-related changes at the neural level of automatic processing of emotions, and the findings are inconsistent. In a combined functional magnetic resonance imaging (fMRI) and event-related potentials (ERP) study by Williams and colleagues [7], no significant age-related changes in the temporooccipital components emerged, suggesting preservation of emotional facial encoding across lifespan. Using a go/no go task with positive, negative, and neutral facial expressions (task irrelevant stimulation), Hilimire et al. [8] found pronounced early negativity at occipital sites and positivity at frontocentral sites to positive emotions in older adults. In young adults, a similar pattern emerged for negative emotions. The authors concluded that aging is characterized by enhanced early processing of positive emotions [8].

Indeed, most research on the aging of emotion processing focused on facial expressions; for example, see [8–11]. Less is known about age-related changes underlying automatic encoding of emotion within the auditory modality and, in particular, their neural correlates. The present study investigated the effect of aging on the neural response of automatic processing of prosody change detection using an oddball paradigm, that is, mismatch responses [12, 13]. In this fMRI variant of mismatch negativity [14, 15], participants were presented with deviant events (emotions and gender neutral prosody) embedded in a stream of standard sounds (female voice with neutral prosody), while they were watching a silent movie [16]. Due to the reported decline of the recognition of negative emotions in aging adults, we studied encoding of negative prosody at early sensory level across different age groups. Although, some studies reported reduced response in the elderly [9, 17, 18] suggesting reduced encoding of negative emotions, others reported no significant difference to negative emotions [19] or novel faces [20]. We hypothesized that responses to negative prosody at the superior temporal gyrus (STG) will decrease over age (hypothesis 1). Positive emotion recognition has been found preserved across aging [6]. According to the positivity bias hypothesis [8], we expected even increasing responses to positive deviants with age (hypothesis 2). Finally, women detected emotional cues better than men [21–23] and their ability to discriminate emotions was preserved with aging [5]. Thus, we hypothesized an age by sex interaction with reduced response amplitudes to prosodic cues in older men compared to women (hypothesis 3).

## 2. Materials and Methods

### 2.1. Participants

Fifty-nine participants were recruited through advertisement in a local newspaper and at RWTH Aachen. Two participants were subsequently excluded due to a low response rate (two or less answers) in the auditory screening test and two more at the participants' request. The participants were recruited for three age groups: young (age range 18–35 years), middle-aged (36–55 years), and older adults (56–75 years). Inclusion criteria were age range 18–75 years, no psychiatric and neurological disorders, no MRI contraindication, normal or corrected to normal visual and auditory acuity, and native German speaker. We used cutoff at the age of 75 because the prevalence of hearing loss increases for older subjects in 50–80% of the population [24]. Also, accumulating MRI contraindications may render the older sample nonrepresentative. Each participant completed a screening test for hearing ability, in which pure tones of 430, 2000, and 4096 Hz were presented to either left or right ear with varying intensity (software Presentation v14.2, http://www.neurobs.com/ [5]). Correct source localization indicated intact hearing. Structured Clinical Interview for DSM-IV German version (SKID-PIT Light [25]) screened for the presence of any Axis-I disorder. Edinburgh Handedness Inventory [26] assessed hand preference. Except for one participant who was ambidextrous, all the others were right-handed. The current affective state was assessed with Positive and Negative Affect Schedule (PANAS [27]) and depressive symptom with the Beck Depression Inventory revised version II (BDI II [28]).

The local ethical committee approved the study and it was performed accordingly to the Declaration of Helsinki. All participants gave written informed consent after receiving a full explanation of the experiment.

### 2.2. Stimuli and Design

Disyllabic pseudowords created following German phonological rules, spoken by one female and one male speech therapists, were selected from a validated database [16], based on accuracy rates (>80%). These pseudowords were spoken with angry, happy, sad, and neutral prosody. Stimuli were normalized to the same peak intensity. We chose happiness as the positive basic emotion and anger as the negative emotion with comparable arousal. The second negative emotion, sadness, was added as low arousal emotion comparable to the neutral condition.

**Figure 1:**
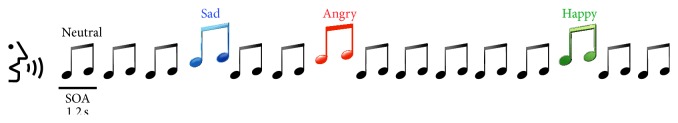
Illustration of the experimental design. In a passive oddball design, standard stimuli were applied, that is, a random dissyllabic utterances from female speakers in a neutral voice. Twenty percent of the stimuli were deviant events, that is, either sad, happy, angry, or male utterances. SOA: stimulus onset asynchrony.

We employed a passive oddball paradigm with 80% standard (frequent) stimuli and 20% deviants. Standard stimuli were pseudowords uttered by female neutral voice. Deviants were pseudowords uttered with either angry, sad, and happy prosody by a female voice or neutral prosody by a male voice (gender deviant). Stimuli were presented binaurally in a randomized sequence, although controlling that one deviant type was not presented twice one after each other and that there were minimum two and maximum nine standards between two deviants. Stimulus onset asynchrony (SOA) was 1.2 seconds (Figure 1). Two runs were conducted in 8 minutes and 80 seconds each with 400 stimuli presented per run. We used Presentation v14.2 (http://www.neurobs.com/) program for stimuli delivery and experimental control. Sound loudness was individually adjusted at the beginning of the scanning. A silent movie was presented during audiostimulus presentation. These movies were cut from a nature documentary (“Earth,” 2007, Disneynature), so that they will have a neutral content. Participants were instructed to pay attention to the movie and to try to ignore the sounds. To ensure that participants will direct their attention toward the movie, they were told that at the end of the scanning they completed a short questionnaire about these movies. Thus, participants were required to rate the emotion induced by these movies using a 5-point Likert-like scale where 1 was very negative, 3 was neutral, and 5 was very positive.

### 2.3. Behavioral Testing

After functional imaging of the oddball paradigm, participants performed a prosodic emotion recognition task employing angry, happy, sad, fearful, disgusted, and neutral utterances. 108 different stimuli were selected from the same database [16] and presented in a random order. Three male and three female speakers were selected, yielding 18 stimuli for each emotional category. Stimulus length was normalized to 700 ms. The interval between two successive stimuli was maximum 8 seconds or until a response was given. Participants selected one of the response keys that best described the emotion uttered. The six emotion labels were continuously displayed on the screen.

Emotion recognition data analysis was performed in SPSS 10.0.0 (SPSS Inc., Chicago, Illinois, http://www.spss.co.in/). Missing responses were excluded from the analysis. Repeated measurement analysis of variance was conducted testing for group effect on the reaction time. Accuracy was a categorical variable (true/false) and analyzed using a Generalized Linear Model (binary response with a probit link function; Wald chi-squared test) with emotion and age-group as predictors. We repeated the analysis examining for a sex effect with sex and age-group defined as between-subject factors and emotion defined as within-subject factor. In case a significant effect was observed, post hoc tests were conducted using Bonferroni correction. The significance level was set to *p* < 0.05 and estimated marginal means (EMM) and standard errors (SE) are reported.

### 2.4. fMRI Data Acquisition and Analysis

Neuroimaging data were acquired on a 3-Tesla MAGNETOM Trio MR Scanner (Siemens, Erlangen, Germany) using a 12-channel head coil. Functional images were acquired in the axial plan using T2^*∗*^-weighted gradient echoplanar image (EPI) with repetition time (TR) being 2000 ms, echo time (TE) being 28 ms, flip angle being 77 degrees, matrix size being 64 × 64, voxel size being 3 × 3 mm, slice thickness being 3 mm, slice gap being 3.75 mm, 34 slices, and field of view being 192 × 192 mm. Two functional runs were conducted and each run comprised a total of 250 volumes. A high-resolution anatomical scan was acquired using a T1-weighted 3D sequence (TE = 2.52 ms; TR = 1900 ms; TI = 900 ms; flip angle = 9°; FOV: 256 × 256 mm²; 1 mm isotropic voxels; 176 sagittal slices).

Prior to analysis, structural and functional data were visually inspected to ensure that no gross artifacts were present. Data preprocessing and analysis were performed using Statistical Parametric Mapping (SPM8, Welcome Department of Cognitive Neurology, UK, http://www.fil.ion.ucl.ac.uk/) implemented in MATLAB 7.10. The first nine volumes of each functional session were discarded to ensure signal stabilization. Functional images were slice timing corrected; realigned to the first volume of the first session to correct for within- and between-sessions motion; coregistered to the anatomical image; normalized into Montreal Neurological Institute (MNI) space using an affine fourth-degree b-spline interpolation transformation, and resliced with a resolution of 3 × 3 × 3 mm. Movement parameters for each participant were inspected using an exclusion criterion of more than 3 mm or 3-degree rotation in any direction. Finally, functional images were spatially smoothed with an 8 mm full width at half maximum Gaussian kernel.

First level analysis employed the general linear model in an event-related design. Each deviant was modeled as a stick function convolved with the canonical hemodynamic response function (HRF) and its temporal derivative (TD) as implemented in SPM8. Separated regressors were created for each deviant type. Standard stimuli were implicitly modeled in the design. Statistical parametric maps for the HRF and the TD were generated using *t*-tests to identify regions activated during each deviant type, that is, anger, happiness, sadness, and gender, relative to the standard (frequent) stimuli.

Second level analysis, first, investigated global changes in response amplitudes with age. Therefore, regression analysis assess tested a linear effect of age on the neural response to the different deviant responses. Second, to investigate nonlinear and hemodynamic effects, the contrasts entered into a mixed-model analysis of variance with deviant type and basis functions (HRF and TD) defined as within-subject factor and age-group defined as between-subjects factor. The factor depicting basis functions was defined as a two-level factor with unequal variance across the levels and sphericity not assumed. Further, we tested for a sex effect employing a two-way analysis of variance with sex and age-group defined as between-subjects factors and basis functions defined as within-subject factor for each deviant type.

Significant threshold for the main effects was set to *p* < 0.05 after family-wise error (FWE) correction for multiple comparisons across the whole brain. *F*-tests assessed interactions of group by deviant type (on HRF only) and group by deviant by basis functions. To test for group effects, the FWE correction was applied to the region of interest (ROI) encompassing bilateral superior temporal lobe including the auditory cortices (bSTL; WFU-Pickatlas [29]).

Voxel-based morphometry implemented in VBM8 toolbox with default parameters controlled for age-related structural changes on differences in hemodynamic responses. The high-resolution T1 images were bias-corrected, tissue classified, and registered using linear (12-parameter affine) and nonlinear transformation (“warping” [30]). The gray matter maps were smoothed with an isotropic Gaussian kernel of 8 mm full width at half maximum. The total brain volume (TBV) was calculated as the sum of gray matter and white matter density extracted from the segmented images and entered as a linear covariate of no interest in the mixed-effect model as described above. Due to group difference on years of education and depressive symptoms, we repeated the mixed-effect model analysis controlling for BDI scores, years of education, and TBV.

## 3. Results

### 3.1. Demography and Neuropsychology

Table 1 displays the characteristics of the groups. A group effect was found on educational level (*F*[2,54] = 19.74, *p* < 0.005), depressive symptoms (*F*[2,53] = 3.52, *p* = 0.04), and brain volume (*F*[2,51] = 3.73, *p* = 0.03). Young adults had more years of education than middle-aged and older adults (*p* < 0.05). Older adults scored higher on BDI and had reduced brain volume than younger adults (*p* < 0.05). No significant group effect was found on mood (global PANAS score: *F*[2,45] = 2.81, *p* = 0.07), handedness: (*χ* ^2^[2, *N* = 55] = 1.93, *p* = 0.38), and gender: (*χ* ^2^[2, *N* = 55] = 0.68, *p* = 0.77). There was no group effect on emotional movie rating (*F*[1,52] = 0.68, *p* = 0.41); independent from age, participant rated the movies as neutral.

### 3.2. Behavioral Data

Behavioral data of six participants were lost because of technical problems related to computer crashes or because the experiment was stopped prior to its completion. Reaction time (RT) and emotion recognition accuracy data partially confirmed the previously published findings [5] and are summarized in Table 2. Significant effects were found for emotion (*F*[5,41] = 26.03, *p* < 0.05) and group (*F*[2,45] = 21.74, *p* < 0.005) on the reaction time. Post hoc test showed that older adults were significantly slower in responding than young and middle-aged adults (all *p* < 0.005). Group by emotion interaction failed significance (*F*[10,84] = 1.59, *p* = 0.12). Repeating the analysis with age and sex between group effects, we found no significant effect of sex (*F*[1,42] = 0.13, *p* = 0.72) or group by sex interaction (*F*[2,42] = 1.37, *p* = 0.26).

Significant effects on accuracy were found for group (*χ* ^2^[2] = 197.04, *p* < 0.005), emotion (*χ* ^2^[5] = 330.12, *p* < 0.005), and group by emotions interaction (*χ* ^2^[10] = 20.53, *p* < 0.05). The main effect of group indicated that in overall emotions young adults performed better than middle-aged and older adults and middle-aged adults performed better than older adults (all *p* < 0.05). The group by emotion interaction indicated that old adults perform worse than young and middle-age adults, for all prosodies except fearful, in which case they performed worse only relative to young adult. All *p* < 0.004; 95% Wald confidence interval [CI]: range from [0.06,0.22] in middle-aged versus older adults for sad to [0.21,0.37] in younger versus older adults for disgusted prosody; Table 2. Significant difference between young and middle-aged adults emerged for happy prosody (*p* = 0.001, 95% Wald CI [0.04,0.15]).

Repeating the analysis including the sex variable, main effects were found for sex (*χ* ^2^[1] = 25.88, *p* < 0.005), group (*χ* ^2^[2] = 158.34, *p* < 0.005), and emotion (*χ* ^2^[5] = 325.45, *p* < 0.005). Significant interactions were found for group by emotion (*χ* ^2^[10] = 30.90, *p* < 0.005), emotion by sex (*χ* ^2^[5] = 27.28, *p* < 0.005), and group by emotion by sex (*χ* ^2^[10] = 28.24, *p* < 0.005), but not for group by sex (*χ* ^2^[2] = 2.71, *p* = 0.26). In post hoc tests, female participants performed better than male participants on recognizing fearful and happy prosody (all *p* < 0.05).  Table 3 displays the accuracy per emotion of age by sex groups. Overall, males and females showed a similar decline of emotion recognition performance with age, except for fearful, neutral, and sad prosody where an effect of sex by age was observed (see Table 3). Within the age group, sex differences were found for fearful and happy prosody, with a significant better performance for females (Table 3).

### 3.3. fMRI Results

**Figure 2:**
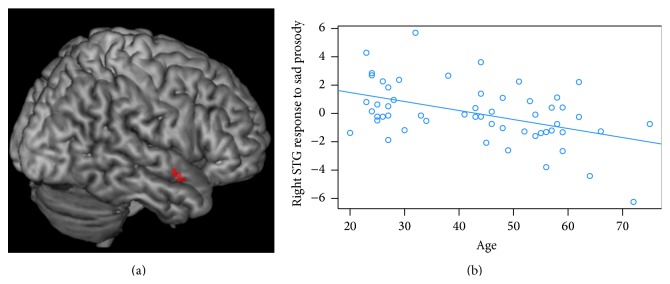
(a) Right STG [*x* = 57, *y* = 2, *z* = −14] response to sad prosody showing a negative correlation with age. (b) The responses at the right STG consistently decrease with age.

Linear-regression analyses revealed a significant negative correlation between age and right STG responses to sad prosody (cluster peak at MNI = [57,2, −14]; cluster size at *k* = 17 voxels; peak at *Z* = 4.11; *p* = 0.016 after FWE correction for bSTL volume; Figure 2). No significant correlation emerged between age and responses to happy, angry prosody, or male voice at this threshold.

In the mixed-effect model, processing of deviants elicited responses at bilateral STG only (right [66, −16,1], *k* = 641, *Z* > 8.0 and left [−60, −10, −2], *k* = 406, *Z* > 8.0, and *p* < 0.05 FWE whole brain correction). Thus, bSTL could be used as a further conservative limitation of the investigated brain volume. A main effect of deviant type emerged in bilateral STG (right [66, −22,1], *k* = 25, and *Z* = 3.88 and left [−54, −7, −5], *k* = 50, *Z* = 4.45, and *p* < 0.05 FEW correction for bSTL). No significant main effect of age groups emerged in this threshold.

**Figure 3:**
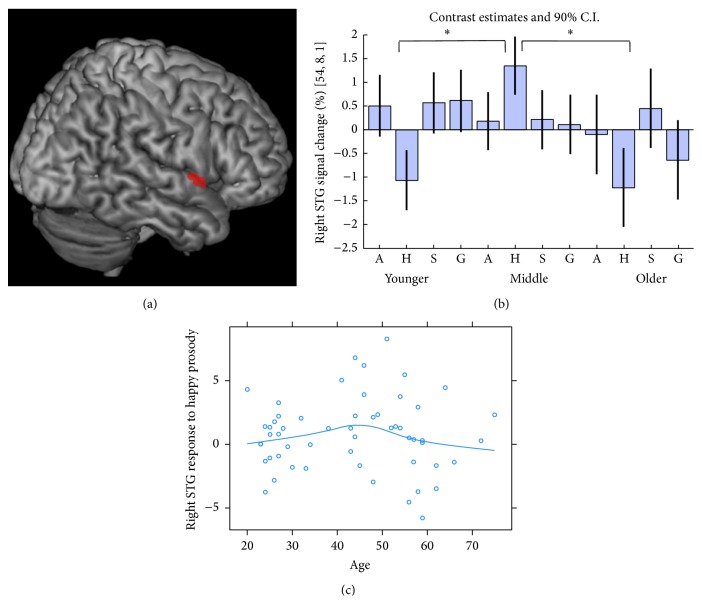
(a) Group by deviant type interaction at the right STG ([*x* = 54, *y* = 8, *z* = 1], *p* < 0.05 few correction for bSTL). (b) Bar plots depict % bold effect and the 90% confidence interval (C.I.), gray lines, in right STG within each group and for each deviant. Stars indicate significant groups differences (*p* < 0.05). (c) The relation between age and right STG response to happy prosody reveals an inverted U-shape. A: angry, H: happy, S: sad, and G: gender (male).

A significant group by deviant type interaction emerged in the right STG ([54,8, 1], *k* = 16, *Z* = 4.15, and *p* < 0.05 FWE correction for bSTL; Figure 3(a)). No brain areas outside bSTL showed significant effects. To further characterize this interaction, *F*-tests determined the group effect within each deviant type. Only for happy prosody, a significant group effect emerged (right STG [57, −13,7], *k* = 34, *Z* = 4.48, and *p* = 0.002, FWE correction for bSTL). In post hoc *t*-tests, responses to happy deviants were larger in middle-aged adults than in young and older adults (*Z* > 4.48, *p* < 0.05; Figure 3(b)). No significant difference on right STG response to happy prosody was found between young and older adults. Indeed, as already suggested by the regression analysis in Figure 3(c), response amplitudes and age seemed to vary in an inverted U-shape fashion.

Further, we investigated if there is a significant group by deviant interaction on the response shape including HRF and time derivate. Bilateral STG yielded a significant group by deviant type by basis function interaction (right [51,2, 1], *k* = 34, *Z* = 4.66; left [−66, −37,19], *k* = 24, *Z* = 4.29, and both *p* < 0.05 in bSTL).

Regarding sex differences, no significant sex by age-group interaction emerged in the STG responses.

Repeating the analysis controlling for age-related structural changes using the total brain volume as covariate of no interest, the effects remained comparable, in particular, the group by deviant interaction at the right STG ([54,8, 1], *k* = 16, *Z* = 4.15). The group by deviants effect was significant, even after controlling for depressive symptom, education (years), and TBV ([57,8, 1], *k* = 23, *Z* = 4.37, and *p*_FWE_ < 0.05 small volume correction), whereas the main effect of deviants was at a trend level (*p*_FWE_ = 0.09 small volume correction).

## 4. Discussion

This study examined age-related neural changes underlying automatic processing of emotional prosody. Our previous behavioral data partially corroborate previous findings of an emotion recognition deficit with aging [5] and further specified a sex by age interaction, for fearful and happy prosody recognition. Regarding the neural correlates of automatic sensory processing, right STG responses to sad deviants decreased linearly with age, whereas responses to happy deviants were maximal between 35 and 50 years of age. These responses emerged in the right STG only and were not affected by the sex of the listener. The sad voice with low arousal may be particularly prone to reflect an age-related decrease in auditory processing. For the other emotions, top-down modulation may introduce mood bias or selective effects. In combination with the differentiated pattern of emotion recognition accuracy, we conclude that early auditory processing reflects only some of the changes affecting the categorization task. In particular, sex effects may affect other neural networks reflecting social cognition or learning history.

Emotion recognition abilities decrease with age. Behavioral data showed a general decline of emotion recognition ability and a slower reaction time with age. Older adults were found significantly less accurate in recognizing angry, sad, disgusted, happy, and neutral prosody than middle-aged and young adults and fearful prosody relative to young adults. These findings are in agreement with previous reports indicating a general emotion recognition deficit with age [1, 5]. Further, we found that females were in general more accurate at recognizing emotions from prosody than males. Considering age with sex interaction, older females performed better than older males in recognizing fearful prosody, and young females had a higher performance in recognizing happy prosody than young males. For the other emotions, both males and females showed a comparable decline of emotion recognition ability with age.

Age-related changes on the neural correlates of sensory acuity have been previously reported. Reduced visual [31–33] and auditory primary sensory areas [34] activation was reported with advance in age. The present study adds to the literature by indicating a modulatory age effect on automatic encoding of prosody. These findings are in line with previous studies form visual modality indicating decreased sensory areas response to emotional stimuli [8, 31–33]. Hilimire and colleagues [8] reported stronger negativity at occipital sites for sad face in young compared to older adults, whereas for happy faces stronger negativity was reported in older adults relative to young adults. Kensinger and Leclerc [35] suggested that automatic emotion processing is preserved with aging, whereas an age effect results in a more controlled emotional processing, such as emotion regulation and emotional memory involving a different neural mechanism showing an effect of age [11]. In our study, employing an event-related oddball paradigm, frontal areas did not emerge. However, auditory responses to sad prosody perception declined like emotion recognition ability with age. Thus, emotion recognition impairment might be related to decline of sensory ability with aging.

The age-related changes may not be specific to arousal or valence. Anger and happiness are emotions with high arousal, whereas sadness and anger are negative emotions. Our findings do not indicate a generalized age effect specific to arousal or valence but rather variations specific to basic emotions, as previously shown for audiovisual emotions in aging [36] and in neurodevelopmental disorders [37]. Valence and arousal may modulate rather higher level of stimulus processing and cognitive control.

The middle part of the STG is associated with “automatic integration” of emotional cues from voices irrespective of the attention focus or task demand [38, 39]. Thereby, the right hemisphere showed higher sensitivity towards prosody perception [39]. In a mismatch paradigm magnetoencephalography study, detection of emotions and gender elicited bilateral mismatch responses in the temporal cortex, including superior, middle, and inferior temporal gyri [16]. An earlier response (about 100 ms poststimulus latency) emerged predominantly in the right hemisphere for emotions detection and not for gender [16]. The present study not only does replicate the previous finding about the relevance of middle STG in sensory processing of emotional prosody, but also revealed an aging effect.

No significant sex by age interaction emerged at the neural level. Conceivably, automatic encoding of emotional prosody declines similarly in males and females with advance in age. Reports on sex differences of neural mechanism of auditory preattentive processing are variable. One study reported no sex difference in the amplitude, latency or duration, and phonetic change detection [40]. Other researchers reported stronger mismatch negative amplitude to emotional versus neutral prosody in young females indicating that females recruit additional processing resources to changes in emotional prosody [22]. The latter authors concluded that sex-related differences emerged at an “early, automatized stage of information processing.” (page 638 [22]). Donges et al. [23] reported a greater sensitive towards positive facial expression in females using an affective priming paradigm in young healthy participants and no sex differences for negative emotions. Thus, it was suggested that females have an enhanced sensitivity towards emotional cues [21]. The lack of sex differences on the neural mechanism of automatic emotional prosody processing might be due to the longer temporal integration window of the fMRI in our study relative to electroencephalography or magnetoencephalography, which were applied in the above mentioned studies. However, the automatic encoding of emotional prosody seems to be overall equally preserved in both females and males across lifetime.

Although the sample size in the present study is similar to previous research, some caution is appropriate regarding the implication of the results due to the limited sample size. Cognitive abilities were not assessed in the current and therefore our interpretation is limited to sensory processing. However, reaction time is considered an index of cognitive abilities [41] and the overall decrease of reaction time parallels the abilities that reduced with age. Due to the set-up of the design, that is, passive oddball, we could not investigate whether prosody during scanning was perceived clearly. The volume of the sounds was individually adjusted, so that each participant could hear the sounds properly during the scanning. The passive oddball paradigm is well established and reflects sound discrimination in the absence of higher cognitive functions, for example, active attention toward the stimuli. We did find a main effect of deviants, as well as deviant by age interaction in the sensory cortex, which indicates that changes in prosody stimuli were encoded at the sensory level.

## 5. Conclusion

This study suggests that automatic encoding of emotional prosody is influenced by age. Although we observed a general decline in emotion recognition with aging, automatic sensory encoding deficit with aging seems to be specific to sad prosody. Indeed, the initial decline of response to happy stimuli was recovered in the elderly. Cognitive control, continuous learning experience, and in particular a positivity bias may interact with a decline of emotion detection across lifespan.

## Figures and Tables

**Table 1:** Demographics and neuropsychology.

	Young adults (18–35 yrs; *n* = 21)	Middle-aged adults (36–55 yrs; *n* = 19)	Older adults (>55 yrs; *n* = 15)
Age	26.62 (3.48)	47.26 (4.86)	61.33 (5.75)
Females (%)	52	53	40
Right-handed (%)	100	96	100
Years of education	17.29 (1.79)	14.68 (3.45)	11.53 (2.72)^*∗*^
BDI^1^	1.14 (1.88)	2.56 (2.64)	3.20 (2.76)^*∗*^
PANAS^2^	19.17 (6.63)	13.47 (5.36)	17.27 (10.03)
Movies' rating	3.5 (0.55)	3.77 (0.83)	3.26 (0.56)
TBV^3^	1.60 (0.17)	1.53 (0.13)	1.47 (0.09)^*∗*^

*Notes*. ^1^Beck Depression Inventory; ^2^Positive Affect and Negative Affect Scale (global score); ^3^total Brain Volume; yrs = years of age. Means (standard deviations) or percentages (%) are presented. Stars (*∗*) indicate significant difference between groups (*p* < 0.05).

**Table 2:** Behavioral data of prosody recognition.

	Young adults (18–35 yrs; *n* = 21)	Middle-aged adults (36–55 yrs; *n* = 19)	Older adults (>55 yrs; *n* = 15)
RT mean ± standard deviation			
RT angry (sec)	2.04 ± 0.40	2.43 ± 0.59	2.91 ± 0.61
RT fearful (sec)	2.44 ± 0.36	2.63 ± 0.50	3.21 ± 0.49
RT disgusted (sec)	2.61 ± 0.50	2.81 ± 0.56	3.42 ± 0.55
RT sad (sec)	2.53 ± 0.47	2.90 ± 0.61	3.63 ± 0.80
RT happy (sec)	1.94 ± 0.40	2.11 ± 0.47	2.68 ± 0.53
RT neutral (sec)	1.84 ± 0.41	2.10 ± 0.57	2.99 ± 0.52
RT angry (sec)	2.04 ± 0.40	2.43 ± 0.59	2.91 ± 0.61
Accuracy estimated marginal mean ± standard error			
All emotions	0.78 ± 0.02	0.72 ± 0.02	0.56 ± 0.03^*∗*^
Angry	0.84 ± 0.02	0.78 ± 0.02	0.61 ± 0.03^*∗*^
Fearful	0.77 ± 0.02	0.69 ± 0.02	0.57 ± 0.03^*∗*^
Disgusted	0.65 ± 0.03	0.56 ± 0.03	0.33 ± 0.03^*∗*^
Sad	0.57 ± 0.03	0.59 ± 0.03	0.45 ± 0.03^*∗*^
Happy	0.88 ± 0.02	0.79 ± 0.02^*∗*^	0.62 ± 0.03^*∗*^
Neutral	0.89 ± 0.02	0.84 ± 0.02	0.69 ± 0.03^*∗*^

*Notes*. RT = reaction time, yrs = years of age. Stars (*∗*) indicate significant difference between groups (*p* < 0.05), such as older adults had reduced accuracy for angry, sad, disgusted, happy, and neutral prosody than young and middle-aged adults, and significant difference between middle-aged and young adults for happy prosody.

**Table 3:** Age by sex interaction of prosody recognition.

Mean accuracy ± standard error	Young adults (18–35 yrs; *n* = 18)		Middle-aged adults (36–55 yrs; *n* = 18)		Older adults (>55 yrs; *n* = 12)	
	Female	Male	Female	Male	Female	Male
Angry	0.88 ± 0.03	0.80 ± 0.03	0.79 ± 0.03^*∗*^	0.77 ± 0.03	0.63 ± 0.06^*∗*^	0.60 ± 0.04^*∗*^
Fearful	0.79 ± 0.03	0.76 ± 0.03	0.76 ± 0.03	0.61 ± 0.04^*∗*^	0.80 ± 0.05	0.49 ± 0.04^*∗*^
Disgusted	0.68 ± 0.04	0.63 ± 0.04	0.55 ± 0.03^*∗*^	0.57 ± 0.04	0.30 ± 0.05^*∗*^	0.34 ± 0.04^*∗*^
Sad	0.62 ± 0.04	0.52 ± 0.04	0.63 ± 0.03	0.53 ± 0.04	0.45 ± 0.06^*∗*^	0.44 ± 0.04
Happy	0.97 ± 0.01	0.81 ± 0.03^*∗*^	0.81 ± 0.03^*∗*^	0.77 ± 0.03	0.72 ± 0.05^*∗*^	0.59 ± 0.04^*∗*^
Neutral	0.85 ± 0.03	0.92 ± 0.02	0.83 ± 0.03	0.86 ± 0.03	0.75 ± 0.05	0.67 ± 0.04^*∗*^

*Notes*. Stars indicate significant differences between age groups (*∗*) and sex (*〈∗〉*; *p* < 0.05). yrs = years of age.
